# Friends with Benefits: The Positive Consequences of Pet-Friendly Practices for Workers’ Well-Being

**DOI:** 10.3390/ijerph19031069

**Published:** 2022-01-19

**Authors:** Ana Junça-Silva

**Affiliations:** Business Research Unit, ISCTE—Lisbon University Institute, 1649-026 Lisbon, Portugal; analjsilva@gmail.com

**Keywords:** pets at work, pet-friendly practices, organizational identification, psychological well-being, subjective well-being

## Abstract

Although there is evidence that pets may help individuals who are facing significant daily stressors, little is known about the benefits of pet-friendly practices for their owners’ well-being. Based on the social exchange theory and on the Rusbult investment model, we argue that organizational pet-friendly practices will be viewed as a source of support from an organization that increases workers’ organizational identification, which in turn will lead to higher levels of psychological well-being and life satisfaction. For this study, 208 working adults answered an online questionnaire. Results from the study showed that the more pet-friendly practices the higher the workers’ organizational identification, which led to higher indices of psychological well-being and life satisfaction. This study contributes to a better understanding of the human–animal interaction and how pets can function as a resource for individuals’ well-being at work.

## 1. Introduction

The number of pets has significantly increased all over the world [1] and many of them are considered family members. Pets are no longer purely a piece of self-defense, or an “alarm” living in the garden; their representation is changing, and their owners are valuing them in such a way that the emotional attachment to them is becoming stronger. Moreover, pets may be a positive presence and a personal resource for their owners while working [2]. Pets at work can help employees and customers with disabilities, promote emotional support, or be a form of companionship [3]. Indeed, pets are being valued for organizational purposes, and this may justify the increased number of organizations adopting pet-friendly practices. For instance, both Google and Amazon allow their workers to bring their pet dogs to work [4] and, since 1999, have adopted a “take your dog to work day” [1]. 

Despite the evidence of the benefits of pets for positive outcomes such as stress reduction, studies exploring the benefits of pet-friendly practices are scarce [5]. The social exchange theory [6], and the Rusbult investment model [7] may support the positive consequences of pet-friendly practices. According to the social exchange theory, organizations with pet-friendly practices may raise workers’ feelings of obligation towards their organization, which in turn may enhance their sense of connectedness with the company, improving individuals’ satisfaction. Plus, the Rusbult investment model states that individuals tend to feel more committed to a relationship when they derive more satisfaction from it, believe they have few desirable alternatives to the relationship, and have invested heavily in the relationship [7,8]. Accordingly, when individuals feel that their organization invests resources and efforts in their relationship, they tend to feel satisfied with it. In turn, commitment will trigger enactment of behaviors that involve forgoing one’s own needs to benefit one’s relationship with the organization [9].

Answering to the call of studies exploring the benefits of pets in the workplace [5], this study aims to contribute to understanding the process through which pet-friendly practices may promote well-being indicators (psychological and subjective well-being–life satisfaction). Therefore, based on the social exchange theory and on the Rusbult Investment Model, we propose that pet-friendly practices will enhance workers’ organizational identification, which in turn will increase their well-being. In other words, organizations that allow their workers to take their pets to work will have happier individuals, because their organizational identification will also be higher. 

## 2. Human-Pet Interaction

Human-pet interaction and bonding is an interspecies relationship that has its roots in pre-history [10]. Animals provide “one highly reliable association in a person’s life … more consistent and reliable than human–human” [11] and hold a “relationship of mutualism” with their owners; i.e., pet owners believe they not only give, but receive, love and affection from their animals [12]. Indeed, pets can create connections through their vivacity and ability to interact with humans, because they are sensitive to the feelings of their owners [13,14].

The importance of pets for human life has received more attention as pets provide individuals with many benefits, such as stress reduction, and increased well-being [15]. The number of individuals with pets has increased, and pets are considered, for many owners, as family members [5]. As the number of pets increases, and as pets take a more central role in the lives of individuals, there is the need to enlarge the research of pets in other domains such as the workplace [5]. 

### 2.1. Pet-Friendly Practices

Although often overlooked, pets interact with organizations in relevant ways. Recently, organizations and managers have acknowledged this, and some of them are becoming pet-friendly. Pet-friendly practices include: allowing the employee to take his/her pet to work; allowing a few days to mourn a pet’s loss; allowing the employee to take part of the day to take the pet to the veterinarian; pet-based performance rewards; canine hotel vouchers; pet daycare assistance. 

These practices are strategies with benefits for individuals, who become more satisfied [16], and for the organization itself, because: it improves employer branding; becomes more integrated within the community; attracts new talents and loyal stakeholders; and at the same time provides workers with healthier working environments [1,17]. For instance, there are organizations that let their workers take their pets to work, e.g., Amazon (Amazon, Seattle, Washington, EUA) or Google (Google, San Francisco, EUA and Ukraine). Amazon even has a “take your pet to work day and Google has a dog park at the Mountain View campus called The Doogleplex, at the EUA. 

By accommodating pets, organizations promote positive effects for workers, since many of them consider their pet to be a family member [18]. Some benefits of taking pets to work are related to the balance between work and nonwork life, where taking them to work is equivalent to not having to worry about leaving them home alone for the entire day, especially if the employee has a senior pet with special needs, or with a disease that regularly requires medication (e.g., diabetes or a heart injury). These benefits are also relevant for the organization. For instance, it is likely that employees who take their pets to work may stay late to finish tasks, because they do not have the worry of going home to take care of their pet [19]. 

### 2.2. The Benefit of Pet-Friendly Practices 

Pets are a source of social and emotional support as they can: transmit feelings of attachment, safety and protection; facilitate social interactions between individuals; improve trust and positive emotions; enhance well-being, perceived organizational support and job satisfaction [20], and reduce stress [21]. Some studies demonstrated that having a pet around increases positive affect and the number of prosocial behaviors [22]. Similarly, Ref. [2] found that the presence of pets at work reduced stress, improved communication, and enhanced social cohesion. Moreover, Ref. [23] stated that in companies where employees may bring their pets to work, problems tend to be addressed openly, employees have more autonomy, with flexibility for breaks, and greater tolerance for failure and errors. Ref. [24] reported that workers who often took their pets to work reported higher work engagement and work-based friendship, and less turnover intentions, compared to those who never took their pet to work. Ref. [25] also reported that the levels of oxytocin produced when workers interacted with pets throughout the working day were similar to levels produced when receiving a massage. Ref. [24] also showed that those who frequently took their pet to work evidenced higher work-related quality of life, general well-being, home-work interface, job-career satisfaction, more control at work, and better perceived working conditions compared to those who never took their pet to work. An experimental study showed that the presence of a dog in a work group increased positive emotions and improved the social and emotional climate [26]. It is likely that these bonds positively impact workers’ well-being, and at the same time, promote the creation of positive work environments [27,28].

### 2.3. The Rusbult Investment Model and the Social Exchange Theory 

The positive effects of pets at work may be supported by two theories: the Rusbult investment model [8], and the social exchange theory [6].

First, the Rusbult investment model proposes that individuals should feel more committed to a relationship when: they derive more satisfaction from it; believe they have few desirable alternatives to the relationship; and have invested heavily in the relationship [7,8]. In other words, the model proposes that satisfaction, alternatives, and investments each uniquely influence relationship commitment. For example, individuals who are highly satisfied with their organizations see few appealing alternatives, and have invested a great deal in the relationship, thus tending to be more committed to their organization [29]. Accordingly, when satisfied, commitment translates into behaviors that involve forgoing one’s own needs to benefit one’s relationship, in this case the employee–employer relationship.

Second, the social exchange theory proposes that individuals behave by weighing the costs and benefits that they expect to receive, either through concrete rewards (pay, goods), or socioemotional ones, e.g., flexibility [6]. These benefits improve the quality of the interactions between employees and employers, and are strengthened when: (a) the costs are fewer than the valued rewards; (b) there is trust between each part regarding their obligations over time; (c) the exchange is perceived to be fair (which implies mutual adherence to the norm of reciprocity); and (d) there is a psychological commitment between each part [30]. Thus, pet owners who are in organizations with pet-friendly practices may feel a greater sense of obligation and commitment to their organization, which may be translated into higher job satisfaction levels. Organizations with pet-friendly practices that support their workers’ lives with pets may trigger the perceived shared values, leading them to feel more identified with the organization and to increased levels of well-being.

Well-being may be conceptualized in two ways: subjective and psychological [31]. Subjective well-being (SWB) has been operationalized as a multifaceted construct, comprising of cognitive and affective components, i.e., satisfaction with life, positive affect, and negative affect [32,33]. It is aligned with hedonia, and relates to feeling good about one’s life and more short-term pleasure-based happiness [34]. It includes two dimensions: cognitive (life satisfaction; refers to judgment processes), and affective (the frequency of positive affect, and the relative absence of negative one; the experience of emotional and mood states) [35,36]. 

On the other hand, psychological well-being (PWB) is a more pervasive indicator of well-being aligned with eudaimonia [37,38]. Eudaimonic theories suggest that not all human desires, even those that lead to pleasure, result in well-being when fulfilled [37]. In other words, PWB is more than the simple attainment of pleasure, and involves a range of areas in an individual’s life, such as the quality of their relationships, self-acceptance, autonomy, personal growth, environmental mastery, and their sense of meaning and purpose in life [38,39]. 

### 2.4. The Mediating Role of Organizational Identification

Although research on the effects of pet-friendly practices on well-being is limited, the relationship between organizational identification (OI) and well-being outcomes is well documented [40]. Organizational identification has been defined as the “perception of oneness with or belongingness to an organization, where the individual defines him/herself in terms of the organization(s) of which s/he is a member” [41], and “when a person’s self-concept contains the same attributes as those in the perceived organizational identity” [42]. It is the importance of the organization to an individual’s self-concept. By internalizing the values and norms of the organization as a part of their self-concept, employees achieve a sense of meaningfulness, and they become more aware of their place in the social world [43]. Employees tend to describe themselves according to their social contexts (i.e., their organizational membership), tend to desire to enhance their self-concept and self-esteem [44], and tend to evaluate their self-worth according to the social standing of their organization [45]. Hence, they are more likely to identify with an organization whose attributes, values, and practices are attractive and similar to their personal ones. Therefore, it is likely that the more pet-friendly practices an organization provides for their workers who own pets the higher the level of their OI.

It has been theoretically argued that a strong sense of shared identity relates to better employee well-being and less stress [46,47,48] argued that OI impacts well-being, because: (1) it helps satisfy important human needs, such as the needs for safety, belonging, self-enhancement, and uncertainty reduction [43]; and (2) employees who identify highly with their group tend to perceive individuals who belong to the same group more positively, leading to more cooperation, better relationships, and higher levels of satisfaction. As a result, employees who identify themselves closely with their organizations should report higher well-being than less identified employees. 

The relationship between OI and well-being is well-documented [47]. For instance [49] showed that managerial control of space led to discomfort and to lower levels of OI, which in turn was negatively related to job satisfaction and well-being. Likewise, Ref. [50] evidenced a direct effect of OI with turnover intention and emotional well-being. Similarly, Ref. [51], demonstrated that OI positively predicted well-being. Ref. [52] also showed a positive direct effect between OI and well-being. More recently, Ref. [53] demonstrated that leadership functions influenced OI and, consequently, well-being. In a similar vein, Ref. [54] showed a positive association among human resources practices and OI, which subsequently enhanced employees’ well-being. 

Thus, based on the empirical findings, on the Rusbult investment model and on the social exchange theory, we expect that pet-friendly practices, by promoting perceived organizational support, will increase organizational identification and this, in turn, will enhance well-being. Accordingly, the following hypothesis was developed.

**Figure 1:**
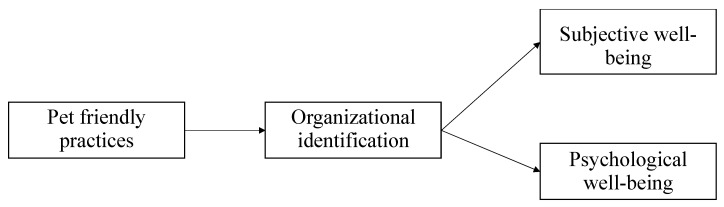
Conceptual model.

**Hypothesis** **1** **(H1).** Organizational identification will mediate the positive relationship between pet-friendly practices and well-being (subjective and psychological) (see Figure 1).

## 3. Method

### 3.1. Participants and Procedure

All participants were Portuguese working adults, and were asked to participate in a study about attitudes at work. They were recruited from the personal and organizational network. Overall, 250 general questionnaires were distributed by email, from which 208 agreed to voluntarily participate in the study (response rate: 83.2%). In the email sent it was mentioned that they should be pet owners, otherwise their participation would not be taken into consideration. 

Most of the participants were female (54%), the mean age was 30.18 years old (*SD* = 10.93), and the mean organizational tenure was 4.67 years (*SD* = 6.22). On average, participants had 2.32 pets (*SD* = 2.46), and the majority of participants were single (37%), or married (26%). Overall, participants had dogs (51%), cats (23%), fish (9%), birds (8%), reptiles (3%), and hamsters (2%). The data collection occurred from June 2020 until September 2020.

### 3.2. Measures

**Pet-friendly practices** were measured by four items based on [2]. These items used “Yes(1)/No(2)” answer options, and included: “I am allowed to bring my pet to work with me,” “I sometimes bring my pet to work with me,” “I sometimes take my pet to work,” and “I sometimes take my pet to the organization.” Cronbach’s alpha was 0.89.

**Organizational identification** was measured with the six-item Organizational Identification Questionnaire [41], e.g., “My organization is a reflection of who I am.” Participants answered on a 5-point scale: (1)–totally disagree; (5)–totally agree. Reliability test indicated acceptable reliability (α = 0.84).

**Life satisfaction** was assessed through the 5-item Satisfaction With Life scale [31], e.g., “In most ways my life is close to my ideal.” Responses were made on a 5-point scale, ranging from 1 (strongly disagree) to 5 (strongly agree). The reliability was good (α = 0.82).

**Psychological well-being** was assessed with the 6-item Ryff Psychological Well-Being scale [38,39], assessing environmental mastery, personal growth, purpose in life, positive relations, self-acceptance, and autonomy, e.g., “I enjoy making plans for the future and working to make them a reality.” Answers were given on a 5-point Likert scale ranging from (1) least like me to (5) most like me. Cronbach’s alpha was 0.70.

**Control variables.** We used sex and age as a control variable. Sex may account for differences in experienced well-being [55], and age may account for differences in the OI [40].

### 3.3. Data Analysis

The hypothesis was tested using PROCESS 3.1 (in SPSS v. 25) (IBM Corporation, Armonk, NY, USA), specifically model 4 (mediation). Control variables (age, sex) were entered, with pet-friendly practices entered as the independent variable, OI as the mediator, and well-being as the dependent. We used bootstrapping (5000 times) to provide confidence intervals. 

## 4. Results

### 4.1. Descriptive Statistics and Correlations

Table 1 shows the descriptive statistics and correlations between the variables. 

### 4.2. Hypothesis Testing

Hypothesis 1 expected that pet-friendly practices would positively influence well-being through OI. First, we tested the model with life satisfaction. The results showed that pet-friendly practices were significantly related to OI (*B* = 0.93, *p* < 0.01, CI 95% (0.26, 1.06)). Furthermore, OI is significantly related to life satisfaction (*B* = 0.31, *p* < 0.01, CI 95% (0.12, 0.48)), and when included in the model, it fully mediated the effect of pet-friendly practices on life satisfaction. The indirect effect was 0.29 with a 95% CI ((0.10, 0.57)), and explained 11% of the variance. 

Second, we tested the model with psychological well-being (PWB). The results showed that OI is significantly related to PWB (*B* = 0.15, *p* < 0.01, CI 95% (0.02, 0.29)), and when included in the model, it fully mediated the effect of pet-friendly practices on PWB. The indirect effect was 0.15 with a 95% CI ((0.02, 0.38)), and explained 5% of the variance. These findings support Hypothesis 1.

## 5. Discussion

The present study examined the role of pet-friendly practices on OI and two well-being indicators. This study answers to the call of studies from [5] to explore the benefits of pets at work for specific outcomes, such as well-being. Specifically, this study aimed to contribute to understanding the process through which taking the pet to work might improve well-being. 

The results show that pet-friendly practices improve OI, which in turn enhances life satisfaction and PWB. In other words, when individuals are allowed to take their pets to work, they become more identified with their organization, and their well-being tends to increase. The social exchange theory and the reciprocity norm [56] help to explain this result. Accordingly, individuals tend to behave by weighing the costs and benefits that they expect to receive, e.g., having days to take their pet to work [6]. Thus, individuals who can take their pet to their working space may feel a sense of obligation towards their organization, and may perceive that their organization shares similar values, increasing the likelihood of incorporating organizational values in their self-concept. By sharing the same values, employees tend to feel their self-concept is more coherent, achieve a sense of meaningfulness, and tend to become more aware of their place in the social world [43]. Moreover, a strong sense of shared identity relates to better employee health and well-being [46,47]. Research generally confirms the viewpoint that positive treatment from the organization (in terms of fairness, job conditions, and quality of employer-employee relationships) results in higher OI from the employee and makes them feel better [40]. Moreover, attachment theory and the conservation of resources theory (COR) help to explain these beneficial effects to workers. Attachment theory [57,58,59] suggests that a close emotional attachment between a pet and an individual provides psychological security, and is a source of social support for the individual, especially during this time of pandemic [60]. In addition, the COR theory [61] helps to explain employees’ personal gains from working with their pets closer. Accordingly, pets may be viewed as a resource for the individual, thereby promoting well-being. Thus, it is likely that OI is boosted when employees are allowed to take their pets to work, and higher OI triggers more frequent feelings of satisfaction and well-being among workers [40]. Although not directed with pet-friendly practices, a recent study showed that human resources management practices positively predicted OI, which in turn improved well-being [54]. 

In this study, we focus on OI as a mechanism to explain how pet-friendly practices impact well-being. These results show that a pet-day may be beneficial, not only for individuals’ well-being, but also for the organization, as individuals more identified with their organization can work better and happier. Thus, including pet-friendly practices in organizational life is a strategy to improve the attitude of employees towards their work, and their subjective and psychological well-being. In other words, take the pets to work and involve them in “office” life is a strategy for those who aim to improve the sharing of values between employee and employer, and the cherry on the top of the cake, is the happiness that it promotes.

### 5.1. Limitations and Future Research

Despite the positive features of this study, such as being a precursory study in a relevant field with a working sample, it has some limitations. First, we used self-reported measures, which might account for common method variance [62,63]. Second, the fact that data were collected from a cross-sectional study is a limitation. OI and well-being must be collected at various time points to understand the existing fluctuations. Therefore, future studies could replicate this study through a longitudinal or daily study. Third, only pet owners were included in this study, which might have biased the findings; pet owners are indeed more likely to find pet-friendly practices of their employer a reason to also value the employer more. However, we do not know whether they are more likely to identify with their organization than non-pet owners, and this could be tested in future studies. Last, only a measure for pet-friendly practices was included, not participants’ attitudes towards those practices. Bringing the pet to work does not necessarily mean that one thinks that is a good, or nice, attribute. Thus, future studies should explore the role of attitudes toward pet-friendly practices, e.g., “I think it is very positive that I can bring my pet to work,” and the impact on diverse outcomes, such as well-being or performance.

These results open the way to future studies. First, the finding that pets at work impact OI and well-being is relevant, as most studies have disregarded the importance of pets for organizational life [5]. Second, it would be interesting to test the model with other criterion variables, for instance overall health or daily symptoms. To do this, future studies could use objective measures of health, such as heart rate or blood pressure. Third, it would be useful to understand the different effects of a service animal, a comfort animal, an emotional support animal, and a pet for health and well-being indicators [64]. Moreover, future studies could consider exploring the differences of each kind of animal for practical purposes, e.g., performance. Fourth, future research could analyze the situational conditions by which pet-friendly practices predict OI and well-being, for instance through micro-daily events at work [65]. Fifth, future studies should explore the role of different pet species, e.g., dogs, or cats, because there are studies that demonstrated that different species had different effects. A study developed in a dentist’s office showed that an aquarium full of fish provided a relaxing climate and made the space calmer [66]. A study at Ferrari revealed that a cat interacting with customers and workers provided little distraction, and at the same time lowered stress levels [67]. Similarly, diverse studies developed in technology companies showed that dogs interacted more, needed other types of caring and attention, but could make the environment more dynamic, creative, and warming [15]. Last, future studies could test the relationship between organizational commitment and job satisfaction in organizations with pet-friendly practices between workers who own pets and those who do not. 

### 5.2. Practical Contributions

This research allows the conclusion that pet-friendly practices are an important variable for the prediction of employees’ well-being. Thus, the relevance of pets at work has important implications for organizational theories and applied purposes, such as performance management, and employee development.

The presence of pets, as well as other practices related to them, seem to be relevant to satisfy the needs of employees and their customers, and at the same time deliver benefits to organizations. It has been argued that the implementation of pet-friendly practices has reduced organizational costs, especially when compared to the advantages it has [68,69]. These benefits assert themselves even in the face of challenges related to health, safety, cultural issues, fears and phobias, and interruptions in the work environment [70,71]. 

However, practices must be implemented in baby steps. For instance, it could be a starting point to create a “pet day”, that is an open day in which workers and customers could take their pet to work. Another measure could include a license of bereavement following a pet death, or allowing the owner to take the day off for their pet’s birthday. Other measures could include the extension of family friendly practices to include pets. For instance, many organizations have aids for their workers’ children’s education. However, it could be extended to pet caring or to pet day care. 

## 6. Conclusions

Overall, this study evidence that individuals who can take their pet to work are more identified with their organizations, and are happier. Thus, “who let the dogs out” triggers organizational identification that accounts for workers’ well-being.

## Figures and Tables

**Table 1:** Correlations and descriptive statistics of the variables.

Variables	*M*	*SD*	1	2	3	4	5	6
1. Pet-friendly practices	1.10 ^1^	0.53	(0.89)					
2. OI	3.45	0.75	0.25 **	(0.90)				
3. Life satisfaction	3.61	0.76	0.10	0.31 **	(0.84)			
4. PWB	4.03	0.56	0.04	0.14	0.44 **	(0.70)		
5. Sex	-	-	0.24 **	−0.05	0.08	0.06	-	
6. Age	30.18	10.93	0.15	0.25 **	0.06	0.16	0.16	-

Note. N = 208; ** *p* < 0.001; Cronbach’s alphas are in brackets. PWB: psychological well-being. OI: Organizational identification. ^1^ Responses to pet-friendly practices were dichotomic (1—yes/2—no).

## Data Availability

Data will be made available upon reasonable request.
